# Performance comparison of different classification algorithms applied to the diagnosis of familial hypercholesterolemia in paediatric subjects

**DOI:** 10.1038/s41598-022-05063-8

**Published:** 2022-01-21

**Authors:** João Albuquerque, Ana Margarida Medeiros, Ana Catarina Alves, Mafalda Bourbon, Marília Antunes

**Affiliations:** 1 Departamento de Biomedicina, Unidade de Bioquímica, Faculdade de Medicina, Universidade do Porto, 4200-319 Porto, Portugal; 2 Centro de Estatística e Aplicações, Faculdade de Ciências, Universidade de Lisboa, 1749-016 Lisboa, Portugal; 3 Grupo de Investigação Cardiovascular, Departamento de Promoção da Saúde e Prevenção de Doenças Não Transmissíveis, Instituto Nacional de Saúde Doutor Ricardo Jorge, 1649-016 Lisboa, Portugal; 4 Instituto de Biossistemas e Ciências Integrativas, Faculdade de Ciências, Universidade de Lisboa, 1749-016 Lisboa, Portugal; 5 Departamento de Estatística e Investigação Operacional, Faculdade de Ciências, Universidade de Lisboa, 1749-016 Lisboa, Portugal

**Keywords:** Computational models, Machine learning, Predictive medicine, Statistical methods, Diagnostic markers, Predictive markers, Disease genetics

## Abstract

Familial Hypercholesterolemia (FH) is an inherited disorder of lipid metabolism, characterized by increased low density lipoprotein cholesterol (LDLc) levels. The main purpose of the current work was to explore alternative classification methods to traditional clinical criteria for FH diagnosis, based on several biochemical and biological indicators. Logistic regression (LR), decision tree (DT), random forest (RF) and naive Bayes (NB) algorithms were developed for this purpose, and thresholds were optimized by maximization of Youden index (YI). All models presented similar accuracy (*Acc*), specificity (*Spec*) and positive predictive values (*PPV*). Sensitivity (*Sens*) and *G*-mean values were significantly higher in LR and RF models, compared to the DT. When compared to Simon Broome (SB) biochemical criteria for FH diagnosis, all models presented significantly higher *Acc*, *Spec* and *G*-mean values (*p* < 0.01), and lower negative predictive value (*NPV*, *p* < 0.05). Moreover, LR and RF models presented comparable *Sens* values. Adjustment of the cut-off point by maximizing YI significantly increased *Sens* values, with no significant loss in *Acc*. The obtained results suggest such classification algorithms can be a viable alternative to be used as a widespread screening method. An online application has been developed to assess the performance of the LR model in a wider population.

## Introduction

Familial Hypercholesterolemia (FH) is an autosomal dominant disorder of lipid metabolism, characterized by increased low density lipoprotein cholesterol (LDLc) levels^1^. The high cholesterol levels from birth lead to its accumulation in arterial walls, promoting the early development of atherosclerosis, which represents a major risk factor for cardiovascular disease (CVD)^1,2^. This disorder can be divided into a heterozygous (HeFH), and a more severe homozygous form (HoFH). Due to the fact that it can go undetected for many years, HeFH is the focus of the present study, and unless stated otherwise, will be referred simply as FH. FH is caused by mutations in three identified genes that encode key proteins involved in the LDL receptor (LDLr) endocytic and recycling pathways, LDL receptor (*LDLR*), apolipoprotein B (*APOB*) and proprotein convertase subtilisin kexin type 9 (*PCSK9*), which account for around 90%, 5–10% and 1–5% of FH cases respectively^3–5^. A small percentage of FH-like phenotype cases is attributed to rare variants in other genes linked to dyslipidaemia, or to a form of polygenic hypercholesterolemia^2,6^. Recent studies suggest worldwide FH prevalence rates to be between 1:200 and 300^7,8^.

The early diagnosis of FH has been associated with a significant reduction in CVD risk, supporting the introduction of adequate and more aggressive therapeutic measures^9^. There are different clinical criteria available for the diagnosis of FH, although only genetic testing can positively confirm the diagnostic. The importance of molecular diagnosis to characterize FH is evidenced by large cohort studies, in which individuals with clinical criteria for FH that are confirmed to have a causative pathogenic variant, present a significant increase in the risk of coronary heart disease compared to clinical FH patients in whom a causative variant is not found^10,11^. However, because molecular diagnosis is costly and time-consuming, it cannot be used as a first line screening method, and previous selection of patients based on more accessible parameters should be performed. Simon Broome (SB) clinical criteria are among the most commonly used in the paediatric population, and are based on LDLc and total cholesterol (TC) levels, family history and presence of tendon xanthomas^4,12^. Since physical signs are a rare finding in individuals at paediatric age^6^, and information concerning family history is frequently absent^13^, the decision regarding a possible FH diagnosis is often made based solely on biochemical criteria. Due to the fact biochemical cut-off values used in SB criteria are very conservative, application of these criteria results in a high false positive rate when compared to molecular study results^6,7^. This issue constitutes a heavy burden in terms of healthcare costs, limiting the access of a larger universe of true FH cases to the genetic study.

There are several algorithms available to handle a classification problem with a binary response variable, in this case representing the positive or negative diagnosis for FH. Logistic regression (LR) is a special case of the generalized linear models methodology. The expected value of the dependent variable given by the logistic function represents the probability of the outcome variable to be FH^14^. The decision tree (DT) model is an algorithm derived from information theory. The classification rule in DT is created by repeatedly dividing the data into increasingly more homogeneous groups, with respect to the variable of interest, a method defined as recursive partitioning^15,16^. The RF algorithm aggregates the results of a multitude of individual DT to classify or predict an observation^17^. Two components of randomness are introduced into the construction of the individual trees. Firstly, each tree is built using a random bootstrapped sample of the training data, a method known as *bagging*^18^. Secondly, a random subset of predictor variables is tested in each of the trees, a procedure designated as the *random subspace selection method*^19^. Naïve Bayes (NB) is a probabilistic classifier based on Bayes’ theorem. This classification algorithm earned the term “naïve” since it relies on the strong, and often erroneous assumption, that predictor features are conditionally independent. Despite its relative simplicity, NB classifier has been shown to outperform even highly sophisticated classification algorithms^20^.

A common way to summarize the results of a classification model is through a confusion matrix, a contingency table where the observed outcome is cross-classified with the predicted outcome. Different operating characteristics (OC) can then be derived, and used to assess the performance of the model^21^. For this purpose, it is necessary to define the cut-off value that best discriminates successes from failures, to use as the classification rule. The default choice of a cut-off value of probability equal to 0.5, is not always the best decision. A particularly useful criterion in clinical diagnostic procedures where the two classes are imbalanced is to maximize *Sens* and *Spec* summation, also known as Youden index (YI). This method attributes equal importance to *Sens* and *Spec* values, ignoring the relative size of the populations^22–24^.

Previous studies have reported an overall good performance of LR^25–28^, RF^28,29^ and DT models^30^ in identifying FH cases. To the knowledge of the authors, there are no published studies assessing the performance of a NB classifier for FH diagnosis. The current study presents however several differences in relation to previous work. Most of the mentioned articles were developed using adult cohorts, while the current paper focuses on the paediatric population. The mentioned studies also have included a vast number of predictor variables routinely available in primary care, while in the current work, fewer parameters, including specific biochemical markers, were included. Another important difference is the fact that ROC curve analysis has been incorporated in the current study, in order to adjust for a cut-off value to optimize *Sens* and *Spec* values, hence addressing the class imbalance problem.

The main purpose of this work was to explore alternative classification procedures for FH diagnosis, based on different biological and biochemical indicators, with improved ability to screen for FH cases in comparison to traditional clinical criteria. The classification algorithms developed for this purpose were LR, NB RF and DT models. For the first three methods, the threshold was further adjusted by maximizing YI, in order to account for class imbalance issues.

## Methods

### Study sample

The sample used in this work was taken from the Portuguese FH study, an ongoing study started in 1999, with the purpose of identifying and characterizing FH in the Portuguese population^31^. 389 observations, corresponding to index patients at paediatric age (2 to 17 years), of both sexes, meeting clinical criteria for hypercholesterolemia (TC ≥ 170 mg/dL or LDLc ≥ 110 mg/dL)^32^, were initially retrieved from the Portuguese FH database. All subjects were white, of European ancestry. From this initial set of data, subjects under hypolipidemic medication at time of biochemical assessment were excluded, together with cases presenting a variant of unknown significance, a monogenic variant in a FH phenocopy gene or HoFH. The final dataset was comprised of 286 paediatric patients, of which 104 had a positive molecular diagnosis for FH. At time of assessment, participants were receiving standard healthcare and nutritional advice. All participants had an informed consent form signed by the legal guardian, and information was registered in a confidential database, approved by the National Data Protection Commission. The study complies with the Declaration of Helsinki and was approved by the ethics committee of the Instituto Nacional de Saúde Doutor Ricardo Jorge (INSA), in Lisbon.

### Candidate predictor and outcome variables

Serum concentrations for a panel of several biochemical variables related to lipid metabolism were used as candidate predictor variables: TC, LDLc, high density lipoprotein cholesterol (HDLc), triglycerides (TG), apolipoproteins AI (ApoAI) and B (ApoB), and lipoprotein(a) (Lp(a)). Concentrations were determined in mg/dL, by enzymatic and colorimetric methods, using a Cobas Integra 400 Plus (Roche) analyser^33^. Additional information, regarding biological and anthropometric variables was also included, specifically age, sex and body mass index z-scores (*z*BMI), calculated according to the World Health Organization (WHO) standards^34,35^. Molecular diagnosis was performed by the study of the *LDLR*, *APOB* and *PCSK9* genes, through fragment amplification by PCR, followed by direct sequencing using Sanger’s method. The promotor, coding and splicing regions of the 18 exons of *LDLR* gene, plus fragments of exons 26 and 29 in *APOB* gene, and five target exons (1, 2, 4, 6 and 9) and flanking regions in *PCSK9* gene were studied, according to the protocol described by Medeiros et al.^36^. Large rearrangements in *LDLR* gene were also searched through MLPA technique. The found variants were classified according to the American College of Medical Genetics and Genomics (ACMG) guidelines^37^. Participants with a positive molecular diagnosis were classified as FH, and participants with a negative molecular diagnosis classified as non-FH.

### Classification algorithms and model comparison

Exploratory and significance analysis of biological and biochemical variables was initially performed for FH and non-FH subjects. Missing values were imputed by means of *k*-nearest neighbours method using Gower’s distance^38^. Variable selection for the LR model was performed by purposeful selection methods, after assessing for collinearity. Continuous predictor variables were previously evaluated for linearity for the *logit*, and when non-linearity occurred, variables were categorized. For the NB model, variables for which significant differences between FH and non-FH patients were verified, and presenting low correlation values were retained. Between highly correlated variables, selection of the variable to retain was made through ROC curve analysis. Continuous variables were log-transformed, whenever a better adjustment to the Normal distribution was provided. The DT was built based on information gain measures, using the *rpart* package from R^16^. The final tree was pruned to avoid overfitting, adopting a complexity parameter (cp) of 0.01. Search for an optimal RF was conducted iteratively, using out-of-bag (OOB) *Acc* estimates for performance comparisons. In a first step, hyperparameter tuning was performed for *ntree*, *mtry* and *node size*, which respectively represent the total number of DT used, the number of predictor variables sampled for each DT, and the amount of pruning in each DT. RF with *ntree* between 10 and 2000, by increments of 10 trees, *mtry* ranging from 1 to 7 variables, and *node size* = 1, 5, 10, 15 and 20 were explored. In a second step, less informative variables were sequentially excluded from the initial set of candidate predictors. The RF model was developed and implemented using the *randomForest* package from R^39^.

A new cut-off value based on YI was calculated for LR, RF and NB models, in opposition to the default cut-off *c* = 0.5. For all classification algorithms, and respective cut-off values, a confusion matrix was generated, by comparison with molecular study results, and different OC were calculated: *Acc, Sens, Spec, PPV, NPV* (negative predictive value) and *G*-mean^21,40^.

**Figure 1:**
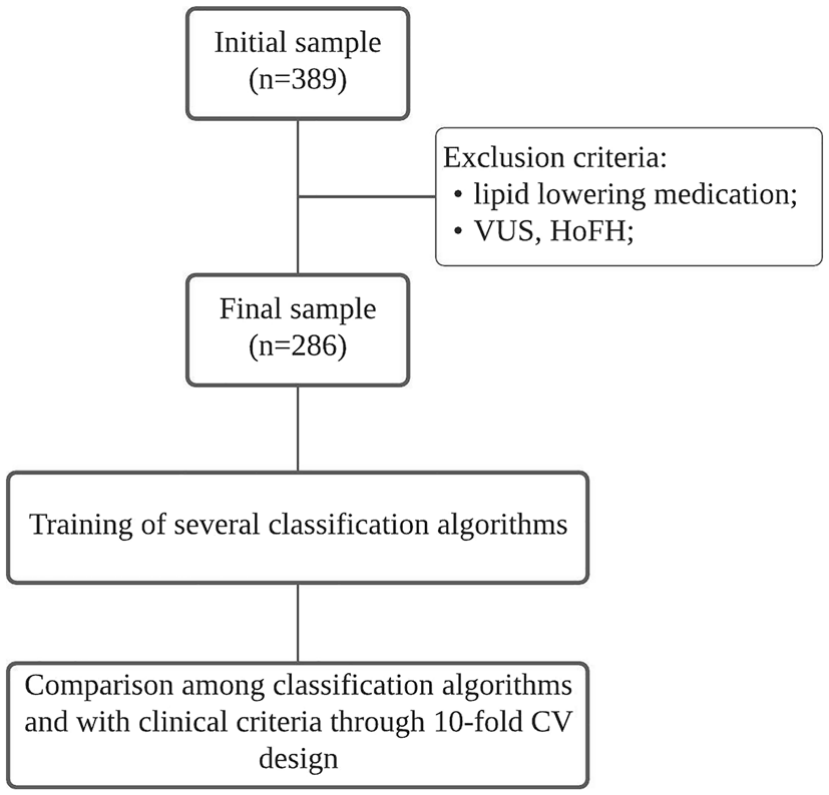
Diagram of overall study design. *VUS* variant of unknown significance, *HoFH* homozygous familial hypercholesterolemia, *CV* cross validation.

Model comparison was performed by means of tenfold cross validation (CV). For each fold, mean values of different OC were calculated (an example of the source code used to test the models is provided on Supplementary Methods S1). Models were compared with SB biochemical criteria (TC > 260 mg/dL or LDLc > 155 mg/dL) regarding these OC, by means of Wilcoxon signed-rank (MWW) test. A diagram of overall study design is represented on Fig. 1. Only biochemical cutpoints in SB criteria were considered, since familial history of elevated cholesterol or CVD is not always available in standard clinical records, and physical signs are very rare to find in paediatric patients^6^. For each OC, different classification algorithms were compared among each other through Friedman test, and in cases where significant differences were observed, pairwise comparison was performed through MWW test. Bonferroni method was used to correct for multiple testing. Statistical analysis was performed using R and R Studio software (v3.5.2), adopting a significance level of α = 0.05.

## Results

### Exploratory analysis

Biological and biochemical characteristics for FH and non-FH patients are presented in Table 1. Significant differences were observed for all predictor variables (*p* < 0.05), except for Age (*p* = 0.19), *z*BMI (*p* = 0.1) and Lp(a) (*p* = 0.17). The 3 non-significant variables were also non-linear with the *logit*, and were therefore categorized. Age was divided into approximately equal range age groups (2–7, 8–12 and 13–17 years), *overweight* was defined as a *z*BMI > 1, following the WHO guidelines^35^, and *high Lp(a)* was defined as Lp(a) serum concentrations above 50 mg/dL, as this cut-off value has been previously established as a risk factor for CVD^41^.

**Table 1:** Biological and biochemical characteristics of FH and non-FH subjects.

	FH	NA %	non-FH	NA %	*p*-value
n (%)	104 (36.4)		182 (63.4)		
n Male (%)	54 (51.9)	0	70 (38.5)	0	0.04
**Age: mean (sd)**	9.36 (3.83)	0	9.9 (3.62)	0	0.19
2–7 years: n (%)	33 (31.7)		40 (22.0)		0.16
8–12 years: n (%)	52 (50.0)		98 (53.8)		
13–17 years: n (%)	19 (18.3)		44 (24.2)		
***z*BMI: mean (sd)**	0.5 (1.2)	6.7	0.76 (1.33)	10.4	0.1
Overweigh: n (%)	33 (31.7)		78 (42.9)		0.08
**Lipid profile (in mg/dL)**					
TC: mean (sd)	272.0 (46.0)	0	230.0 (33.0)	0	< 0.01
LDLc: mean (sd)	203.6 (44.0)	0	153.4 (27.7)	0	< 0.01
HDLc: mean (sd)	52.0 (12.5)	0	59.9 (15.6)	0	< 0.01
TG: mean (sd)	73.2 (32.8)	0	91.8 (43.4)	0	< 0.01
ApoAI: mean (sd)	134.7 (22.3)	2.9	155.1 (27.8)	2.7	< 0.01
ApoB: mean (sd)	133.0 (28.0)	2.9	101.0 (25.0)	1.6	< 0.01
Lp(a): mean (sd)	38.1 (40.6)	10.6	56.1 (65.7)	5.5	0.17
*High Lp(a)*: n (%)	21 (20.2)		74 (40.7)		< 0.01

*FH* familial hypercholesterolemia, *NA* not available, *BMI* body mass index, *TC* total cholesterol, *LDLc* low density lipoprotein cholesterol, *HDLc* high density lipoprotein cholesterol, *TG* triglycerides, *Apo* apolipoprotein, *Lp(a)* Lipoprotein(a), *sd* standard deviation.

Around 36% of the sample presented a positive molecular diagnosis for FH, the great majority with an identified pathogenic variant in the *LDLR* gene (94%), and remaining subjects in *APOB* gene. FH subjects presented higher values for TC, LDLc, and ApoB, and lower values for HDLc, TG, ApoAI and Lp(a) (*p* < 0.05). Missing values were detected for the variables *z*BMI, ApoAI, ApoB and Lp(a), with a percentage of missing cases ranging from 2 to 10%.

### Trained classification algorithms

Final model fit for the LR model is presented in Table 2.

**Table 2:** Final model fit for LR model.

	*β*_*j*_	SE	Wald	*p*-value	OR	95% CI
(Intercept)	− 0.45	1.52	− 0.30	0.77	0.64	(0.03–12.51)
LDLc	0.05	0.01	7.10	< 0.01	1.05	(1.03–1.06)
TG	− 0.02	0.01	− 3.72	< 0.01	0.98	(0.97–0.99)
ApoAI	− 0.04	0.01	− 4.67	< 0.01	0.96	(0.95–0.98)
*Overweight*	− 0.89	0.40	− 2.24	0.02	0.41	(0.18–0.88)
Male sex	0.74	0.37	2.01	0.04	2.09	(1.03–4.33)
*High Lp(a)*	− 0.73	0.39	− 1.86	0.06	0.48	(0.22–1.03)

*SE* standard error, *OR* odds ratio, *CI* confidence interval, *LDLc* low density lipoprotein cholesterol, *TG* triglycerides, *Apo* apolipoprotein, *Lp(a)* Lipoprotein(a).

The final LR model included the continuous variables LDLc, TG and ApoAI, and the categorical variables *overweight*, sex and *high Lp(a)*. Variable selection was the same as the one obtained for NB model, except the fact the *log* of continuous variables was adopted in the latter case, since better adjustment to Normal distribution has been shown.

**Figure 2:**
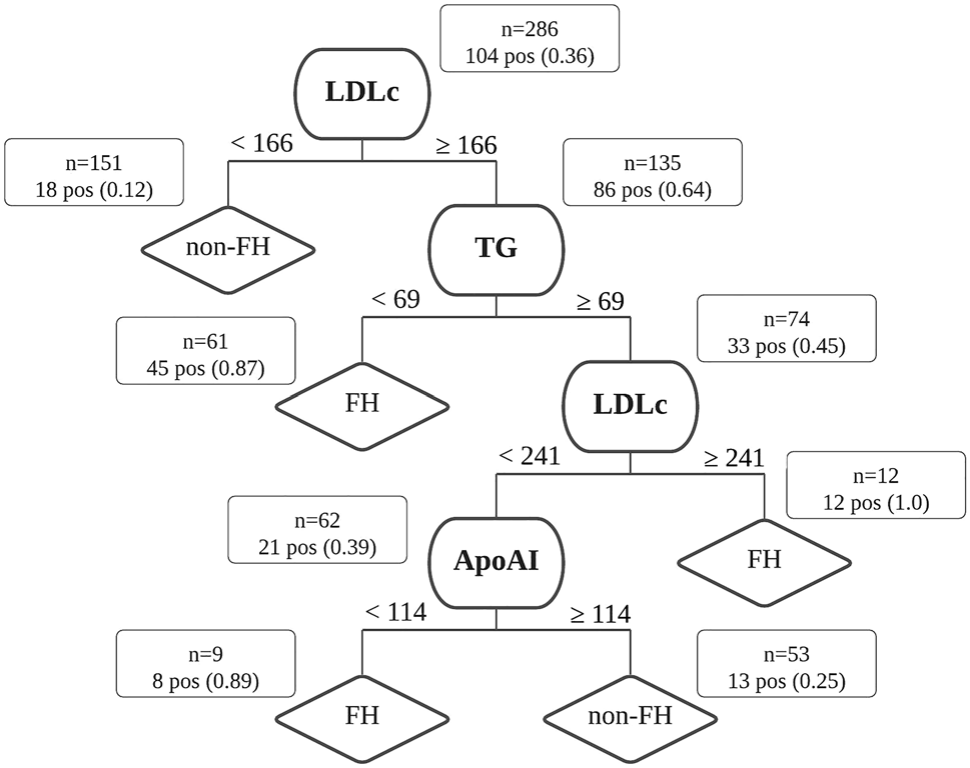
Decision tree model. At each node, it is represented the biochemical indicator used to divide the sample, the respective cut-off value, and the way the original sample is divided throughout the tree. *FH* familial hypercholesterolemia, *LDLc* low density lipoprotein cholesterol, in mg/dL, *TG* triglycerides, in mg/dL, *ApoAI* apolipoprotein AI, in mg/dL, *pos* positive cases.

A representation of the pruned DT model, with respective cut-off values at each node, and showing the tree performance in the complete learning set, is shown in Fig. 2.

Regardless of chosen *mtry*, RF performance seemed to stabilize from around 700 *ntree*. Since increasing the number of trees does not deteriorate RF predictive ability, and computational costs are not very high for the used sample size and number of predictors, a *ntree* = 1000 was defined. The model seemed to achieve better performance without inclusion of variables with very small information loss values (≈1), hence the final RF included only six candidate predictors: TC, LDLc, HDLc, TG, ApoAI and ApoB. For this RF model, optimal *mtry* = 3 and *node size* = 1 were defined.

### Model comparison

**Figure 3:**
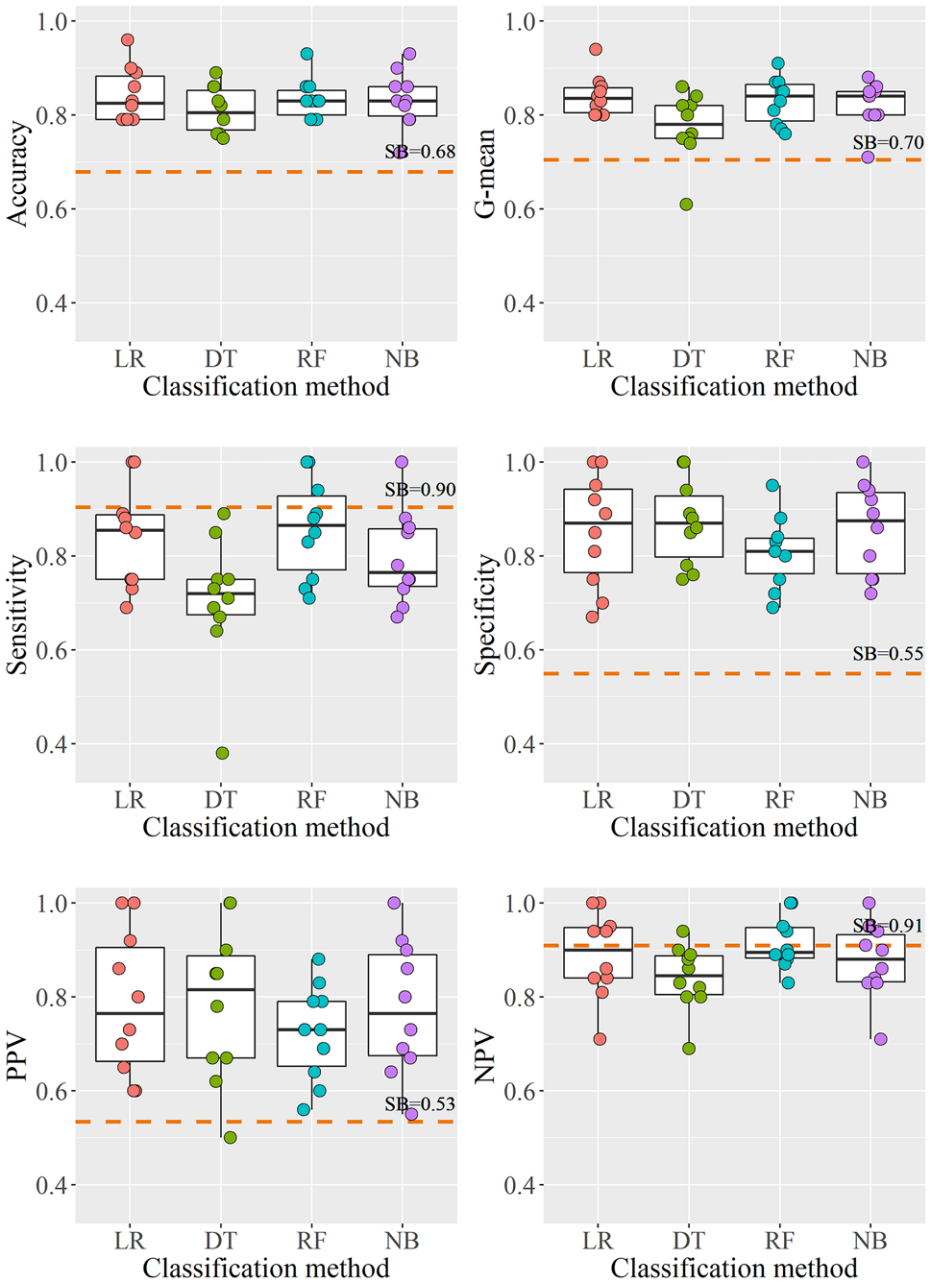
Tenfold cross validation results concerning the several OC, for all classification algorithms. The dashed line represents the value obtained using Simon Broome (SB) biochemical criteria. *LR* logistic regression, *DT* decision tree, *RF* random forest, *NB* Naïve Bayes.

The performance of the different classification models regarding the several OC, as obtained by tenfold CV, together with values that result from the application of SB criteria to the entire sample, are represented in Fig. 3. Corresponding mean values obtained for the different OC by each classification method can be consulted in Supplementary Table S1.

Results concerning OC comparison between pairs of classification algorithms for which significant differences were found, as well as between each classification algorithm and SB criteria, are presented in Table 3.

**Table 3:** Pairwise comparisons tests among different classification methods, and between these and SB criteria, regarding the several OC.

	*Acc*	*G-*mean	*Sens*	*Spec*	*PPV*	*NPV*
LR–DT	*p* = 0.07	*p* = 0.03	*p* = 0.04	*p* = 0.57	*p* = 0.81	*p* = 0.01
RF–DT	*p* = 0.11	*p* = 0.01	*p* = 0.01	*p* = 0.08	*p* = 0.31	*p* < 0.01*
SB–LR	*p* < 0.01*	*p* < 0.01*	*p* = 0.10	*p* < 0.01*	*p* < 0.01*	*p* = 0.91
SB–DT	*p* < 0.01*	*p* = 0.02	*p* < 0.01*	*p* < 0.01*	*p* < 0.01*	*p* = 0.01
SB–RF	*p* < 0.01*	*p* < 0.01*	*p* = 0.26	*p* < 0.01*	*p* < 0.01*	*p* = 0.84
SB–NB	*p* < 0.01*	*p* < 0.01*	*p* = 0.02	*p* < 0.01*	*p* < 0.01*	*p* = 0.29

*Acc* accuracy, *Sens* sensitivity, *Spec* specificity, *PPV* positive predictive value, *NPV* negative predictive value, *LR* logistic regression, *DT* decision tree, *RF* random forest, *NB* naive Bayes Non reported pairwise comparisons did not present any significant difference for *p* < 0.05.

*Still significant for *p* < 0.05 after applying Bonferroni correction for multiple comparisons.

Finally, in Table 4, the mean OC values obtained by the chosen YI method are compared to the ones obtained using the default cut-off value *c* = 0.5, for each classification method.

**Table 4:** Comparison of operating characteristics mean values, as obtained using the default cut-off value c = 0.5 or the value obtained by maximizing YI, among each classification method.

	LR			RF			NB		
	*c* = 0.5	*c* = YI	*p*-value	*c* = 0.5	*c* = YI	*p*-value	*c* = 0.5	*c* = YI	*p*-value
*Acc*	0.84	0.84	0.83	0.84	0.83	0.67	0.84	0.83	1.0
*G*-mean	0.81	0.84	0.20	0.80	0.83	0.13	0.80	0.82	0.11
*Sens*	0.75	0.84	0.04	0.71	0.86	0.01*	0.70	0.79	0.02
*Spec*	0.90	0.85	0.06	0.91	0.81	0.01*	0.92	0.86	0.02
*PPV*	0.82	0.79	0.06	0.85	0.72	0.02	0.84	0.77	0.02
*NPV*	0.86	0.90	0.04	0.84	0.91	0.01*	0.84	0.88	0.02

*YI* Youden index, *Acc* accuracy, *Sens* sensitivity, *Spec* specificity, *PPV* positive predictive value, *NPV* negative predictive value, *LR* logistic regression, *RF* random forest, *NB* naive Bayes.

*Still significant for *p* < 0.05 after applying Bonferroni correction for multiple comparisons.

## Discussion

Four different classification algorithms were implemented and tested in the current study: LR, DT, RF and NB. The cut-off point for three of these models was further adjusted through ROC curve analysis, and defined as the value which maximized YI. This process was not applied to the DT algorithm, since very few candidate cut-points are provided by this model. Due to the fact YI maximization method attributes equal importance to *Sens* and *Spec*, it is considered particularly useful in classification problems in which the two classes are imbalanced^24^. This was the case in the current study, and also in most clinical diagnostic procedures, since the occurrence of a given disease is generally a relatively rare event. Models were posteriorly tested through tenfold CV.

When comparing the different models to each other (Table 3), significant differences were found in *G*-mean, *Sens* and *NPV* values, with the DT model presenting significantly lower values than the LR and the RF models regarding these OC (*p* < 0.05). Since the DT will return as splitting rule the cut-off point that decreases the total entropy of the system the most, it will implicitly favour the majority class, thus decreasing *Sens* and *NPV* values. Several techniques have been suggested to deal with this class imbalance problem, ranging from data sampling methods, to algorithmic modifications, to cost-sensitivity learning^42^. Further work is being prepared incorporating some of these features.

When compared to SB biochemical criteria, all classification algorithms presented significantly higher *Acc*, *G*-mean, *Spec* and *PPV* (*p* < 0.01). This means that overall classification error is smaller, and also that individuals with a positive screening following these methods are more likely to truly be FH. On the other hand, the DT and NB models revealed significantly lower *Sens* levels than SB criteria (*p* < 0.05). Elevated *Sens* in SB criteria is essentially due to very conservative cut-off values, which results in a high number of false positive cases^7^. Reducing the total number of potential candidates to be submitted to molecular diagnosis can have important repercussions in terms of the process cost-effectiveness, and should therefore be taken into account.

Previous studies applying these classification algorithms to FH diagnosis have reported an overall good performance in identifying FH cases^25–30^. Specifically, Niehaus et al.^28^ have studied the performance of a RF against a LR model for FH diagnosis in adults, finding an improved *AUC* (0.91 vs 0.82), *Sens* (0.61 vs 0.56) and *Spec* (0.96 vs 0.91) value for the RF model. One of the potential factors that can account for not observing such differences in the current study, is the fact relatively few predictor variables are used in this work, therefore decreasing the variability of the constituent trees of the RF. Additionally, although overall performance for LR and RF models was similar to the one observed in previous studies^25–29^, as assessed by the *AUC*, *Sens* levels were higher in the current work, and *Spec* values more reduced. Such differences are attributed to the fact that cut-off values in the current study have been defined by maximizing YI, which will make the cutpoint invariably lower, increasing *Sens* and decreasing *Spec* values. An important observation that arises from these results is that it is possible to keep overall *Acc* of a given classifier, while increasing *Sens* levels significantly (Table 4). This is particularly relevant in a clinical screening procedure, where besides obtaining a small classification error, is very important to retain as much disease-positive cases as possible.

Another interesting finding in the current work is the fact that, for all classification algorithms, the most relevant variables selected to classify the individual seem to be LDLc, followed by TG and ApoAI. While LDLc concentration was directly related to the probability of being FH positive, TG and ApoAI levels were inversely associated with the presence of this disorder. While the effect of FH on LDLc metabolism has been extensively studied^1–7,9^, fewer studies that have investigated the relation of this genetic disorder with TG^43^, and ApoAI or HDLc^44^ levels, support the lower levels of these biomarkers found FH individuals. A thorough discussion of the variables included in this study from a metabolic perspective is being addressed in a separate article.

In sum, any of the tested classification algorithms presented higher *Acc*, *Spec* and *PPV* when compared to SB biochemical criteria (*p* < 0.01). Additionally, LR and RF models presented similar *Sens* values than SB biochemical criteria, revealing the ability to detect the same rate of FH cases, with much less false positive retention. An improved *AUC* for LR model, in relation to RF, may favour the selection of this algorithm as a tool for FH classification in the paediatric population. In practice, this means that for a given laboratory annual budget p.e., we would obtain a significantly higher number of cases with a confirmed molecular diagnosis of FH, which would allow adjusting pharmacotherapy, and optimizing subsequent screening measures, by means of cascade screening. As for patients that are classified as non-FH, although it remains possible that a small percentage might carry a causative monogenic variant, as already happens with established clinical criteria, a large proportion of these individuals may have a form of polygenic hypercholesterolemia^6^, or dyslipidaemia triggered essentially by environmental factors, which as already stated, have a significantly lower associated CVD risk^10,11^.

A pilot internet application, implementing LR algorithm, with cut-off values defined by maximizing YI, can be consulted at https://fhidtool.shinyapps.io/dyslipid/. This application was developed using R Studio Shiny package, and named FH.ID.Tool. Paediatricians that collaborate with the Portuguese FH study will be encouraged to use and provide comments to improve the application utilities and interface to a final version, that will then be proposed for implementation at the national health system. Future directions of this work will comprise the development of classification models for the Portuguese adult population, validation of such models on national FH studies of different populations, and study of the relevance of additional biomarkers for FH classification.

## Conclusions

Several conclusions can be taken from the current study, regarding the performance of the different classification algorithms. Higher *Sens* and *G*-mean values were achieved with the LR and RF models, while the DT model was the one that presented lowest values regarding these OC (*p* < 0.05). No significant differences were observed between methods regarding *Acc*, *Spec* and *PPV* values. In comparison with SB biochemical criteria, all classification models presented significantly higher *Acc*, *Spec* and *PPV* (*p* < 0.01). However, even after correction for multiple comparisons, the DT model still presented lower *Sens* values (*p* < 0.01). Low *Sens* values can be a problem in a screening procedure like the one presented in the current study, where retaining the highest possible percentage of FH cases is a major concern. Regarding this matter, cut-off values can be adjusted, based on ROC curve analysis, in order to increase *Sens* significantly, while maintaining *Acc* levels. Based on the results of the current study, LR may be considered the most parsimonious model, when accounting for all OC. The implementation of the proposed LR algorithm as a screening tool will allow retaining a similar number of FH paediatric cases to the clinical diagnosis criteria, while excluding a significant amount of false positive cases. The widespread implementation of such a tool may be beneficial in terms of cost-effectiveness.

## Supplementary Information

Supplementary Information.
