# Supplementary material for: Performance comparison of different classification algorithms applied to the diagnosis of familial hypercholesterolemia in paediatric subjects
Source: Sci Rep. 2022 Jan 21;12:1164. doi: 10.1038/s41598-022-05063-8 (PMC8782861; doi:10.1038/s41598-022-05063-8)
Supplement: Supplementary file 1 — Supplementary Information. [file 41598_2022_5063_MOESM1_ESM.docx]

Supplementary Information

Performance comparison of different classification algorithms applied to the diagnosis of familial hypercholesterolemia in pediatric subjects

João Albuquerque^1,2*^; Ana Margarida Medeiros^3,4^; Ana Catarina Alves^3,4^; Mafalda Bourbon^3,4^; Marília Antunes^2,5^

1 - Departamento de Biomedicina, Unidade de Bioquímica, Faculdade de Medicina da Universidade do Porto, Portugal

2 - Centro de Estatística e Aplicações, Faculdade de Ciências da Universidade de Lisboa, Portugal

3 - Grupo de Investigação Cardiovascular, Departamento de Promoção da Saúde e Prevenção de Doenças Não Transmissíveis, Instituto Nacional de Saúde Doutor Ricardo Jorge, Lisboa, Portugal

4 - Instituto de Biossistemas e Ciências Integrativas, Faculdade de Ciências da Universidade de Lisboa, Portugal

5 - Departamento de Estatística e Investigação Operacional, Faculdade de Ciências da Universidade de Lisboa, Portugal

*Corresponding author: João Albuquerque

Address: Alameda Prof. Hernâni Monteiro, 4200-319 Porto, Portugal

E-mail: [joaodavid.alb@gmail.com](mailto:joaodavid.alb@gmail.com)

Supplementary Methods S1. Example of code for operating characteristics calculation through a 10-fold cross validation process, using the logistic regression model.

## CV process for LR model

# Randomly shuffle the data
set.seed(12345)
Data <- model.data.imp[sample(nrow(model.data.imp)),]

# Create 10 equally size folds
folds <- cut(seq(1,nrow(Data)),breaks=10,labels=FALSE)
Data <- cbind(Data, folds)

# Perform 10 fold cross validation
youden.cutoff <- c()
auc <- c()
accuracy1 <- c()
accuracy2 <- c()
sens1 <- c()
sens2 <- c()
spec1 <- c()
spec2 <- c()
ppv1 <- c()
ppv2 <- c()
npv1 <- c()
npv2 <- c()

for(i in 1:10){
 trainData <- subset(Data, Data$folds != i)
 testData <- subset(Data, Data$folds == i)
 model <- glm(Final.Result ~

Lipids.LDLc + Lipids.TG + Lipids.ApoAI + zbmi.cat + Gender + lpa.cat,
 family = binomial(link = "logit"), data = trainData)

 cut.roc <- ROC(form=formula(model), plot=F, las=1, main="",

data=trainData, MI=F, MX=T)
 prob <- predict.glm(model, newdata=testData, type="response")

auc[i] <- cut.roc$AUC

 opt.cut1 <- 0.5
 opt.cut2 <- cut.roc$res[,"lr.eta"]

[which.max(rowSums(cut.roc$res[, c("sens", "spec")]))]
 youden.cutoff[i] <- opt.cut2
 prob <- predict.glm(model, newdata=testData, type="response")

 res1 <- as.factor(ifelse(prob > opt.cut1, "Positive_htz", "Negative"))
 res2 <- as.factor(ifelse(prob > opt.cut2, "Positive_htz", "Negative"))

 comp1 <- (ifelse(testData$Final.Result==res1, "Same", "Dif"))
 comp2 <- (ifelse(testData$Final.Result==res2, "Same", "Dif"))

 accuracy1[i] <- length(comp1[comp1=="Same"])/nrow(testData)
 accuracy2[i] <- length(comp2[comp2=="Same"])/nrow(testData)

 sens1[i] <- length(comp1[comp1=="Same" & testData$Final.Result=="Positive_htz"])/
 nrow(testData[testData$Final.Result=="Positive_htz"])
 sens2[i] <- length(comp2[comp2=="Same" & testData$Final.Result=="Positive_htz"])/
 nrow(testData[testData$Final.Result=="Positive_htz"])

 spec1[i] <- length(comp1[comp1=="Same" & testData$Final.Result=="Negative"])/
 nrow(testData[testData$Final.Result=="Negative"])
 spec2[i] <- length(comp2[comp2=="Same" & testData$Final.Result=="Negative"])/
 nrow(testData[testData$Final.Result=="Negative"])

 ppv1[i] <- length(comp1[comp1=="Same" & testData$Final.Result=="Positive_htz"])/length(res1[res1=="Positive_htz"])
 ppv2[i] <- length(comp2[comp2=="Same" & testData$Final.Result=="Positive_htz"])/length(res2[res2=="Positive_htz"])

 npv1[i] <- length(comp1[comp1=="Same" & testData$Final.Result=="Negative"])/
 length(res1[res1=="Negative"])
 npv2[i] <- length(comp2[comp2=="Same" & testData$Final.Result=="Negative"])/
 length(res2[res2=="Negative"])
}

# CV results for LR
lr.accuracy <- as.data.frame(cbind(accuracy1, accuracy2))
lr.sens <- as.data.frame(cbind(sens1, sens2))
lr.spec <- as.data.frame(cbind(spec1, spec2))
lr.ppv <- as.data.frame(cbind(ppv1, ppv2))
lr.npv <- as.data.frame(cbind(npv1, npv2))
lr.Gmeans1 <- sqrt(sens1*spec1)
lr.Gmeans2 <- sqrt(sens2*spec2)
lr.Gmeans <- as.data.frame(cbind(lr.Gmeans1, lr.Gmeans2))

lr.cv <- round(cbind(accuracy1, accuracy2, lr.Gmeans1, lr.Gmeans2, sens1, sens2, spec1, spec2, ppv1, ppv2, npv1, npv2, auc), 2); lr.cv

Supplementary Table S1. Mean and standard deviation values of the OC obtained with each model, regarding 10-fold cross validation results.

|  | *Acc* | *G-*mean | *Sens* | *Spec* | *PPV* | *NPV* | *AUC* |
| --- | --- | --- | --- | --- | --- | --- | --- |
| LR | 0.84 (0.06) | 0.84 (0.04) | 0.84 (0.11) | 0.85 (0.12) | 0.79 (0.15) | 0.90 (0.09) | 0.91 |
| DT | 0.81 (0.05) | 0.78 (0.07) | 0.70 (0.14) | 0.87 (0.09) | 0.78 (0.17) | 0.84 (0.07) | 0.87 |
| RF | 0.83 (0.04) | 0.83 (0.05) | 0.86 (0.10) | 0.81 (0.08) | 0.72 (0.10) | 0.92 (0.06) | 0.89 |
| NB | 0.83 (0.06) | 0.82 (0.05) | 0.79 (0.10) | 0.86 (0.10) | 0.77 (0.14) | 0.88 (0.08) | 0.90 |
| SB | 0.68 | 0.70 | 0.90 | 0.55 | 0.53 | 0.91 | - |

*Acc*: accuracy; *Sens*: sensitivity; *Spec*: specificity; *PPV*: positive predictive value; *NPV*: negative predictive value; *AUC*: area under the curve obtained from test observations; LR: logistic regression; DT: decision tree; RF: random forest; NB: naive Bayes; SB: Simon Broome criteria.
